# Combined aerobic and resistance training: are there additional benefits for older hypertensive adults?

**DOI:** 10.6061/clinics/2017(06)06

**Published:** 2017-06

**Authors:** Leandra G. Lima, José T.M. Bonardi, Giulliard O. Campos, Rodrigo F. Bertani, Luria M.L. Scher, Júlio C. Moriguti, Eduardo Ferriolli, Nereida K.C. Lima

**Affiliations:** Departamento de Medicina Interna, Divisao de Clinica Medica Geral e Geriatria, Faculdade de Medicina de Ribeirao Preto, Universidade de Sao Paulo, FMRP-USP, Ribeirao Preto, SP, BR

**Keywords:** Aerobic Exercise, Isometric Exercise, Ambulatory Blood Pressure Monitoring, Aged

## Abstract

**OBJECTIVES:**

The objective of this study was to compare the effects of a combination of aerobic and resistance training to those of isolated aerobic training on blood pressure, body composition, and insulin sensitivity in hypertensive older adults.

**METHOD:**

Forty-four patients were randomly assigned to the aerobic group, resistance and aerobic group, and control group. Before and after 10 weeks, the following data were obtained: 24-hour ambulatory blood pressure data, abdominal circumference, waist circumference, body mass index, lean mass, fat mass, and insulin sensitivity. The study was conducted with 3 training sessions per week.

**RESULTS:**

Comparison revealed significant reductions in the body mass index, abdominal and waist circumferences, and ambulatory blood pressure (24-hour, wakefulness and sleep systolic/diastolic blood pressures) in both the aerobic group and the resistance and aerobic (combined) group. The fat mass only changed in the combined group. There was no difference in the insulin sensitivity in any group.

**CONCLUSIONS:**

The combined treatment and aerobic treatment alone were equally effective in reducing the blood pressure, body mass index, and abdominal and waist circumferences, although the addition of the resistance component also helped reduce the fat mass.

## INTRODUCTION

Aging is a natural, irreversible and individual biological process that is always accompanied by progressive changes 1. However, age is one of the most important factors associated with the increased prevalence of hypertension and insulin resistance. Older adults have greater abdominal adiposity and a higher prevalence of obesity and sedentarism, which are considered factors that elevate the morbidity and mortality of this population.

Good physical ability is important for the autonomy of older adults. Physical ability is related to the production of energy needed for the metabolic processes, which helps reduce the incidence of cardiovascular diseases, especially in hypertensive individuals 2, for whom insulin resistance and/or hyperinsulinemia have been suggested to be responsible for the increase in blood pressure (BP) 3.

While aerobic exercises have been extensively indicated for treating hypertension 4, there are debates in the literature about the hypotensive results of resistive exercises for hypertensive older adults. The acute and chronic effects of resistive exercises are uncertain, suggesting that they are not recommended for the treatment of hypertensive patients alone 5.

In contrast, the benefit of physical exercise on insulin sensitivity has been demonstrated in terms of both aerobic and resistive exercise 6-8. The mechanisms by which resistive and aerobic exercises affect some variables of metabolic syndrome, such as insulin resistance, glucose intolerance and obesity, seem to differ 9,10, suggesting that the combination of the two exercise modalities may have an additive effect.

The effects on body composition reported in studies using different types of exercise appear to diverge. However, the many benefits of aerobic exercise and resistance training are universally accepted, acting in a preventive and compensatory manner on the muscle and body changes involved in primary and secondary aging.

Within this context, we proposed that a program of physical activity for hypertensive older adults should include aerobic and resistive components that might improve cardiorespiratory and hemodynamic conditioning, insulin sensitivity, and body composition.

Because the benefit of aerobic training for hypertensive persons has been well established, the objective of the present study was to assess the effects of aerobic training plus resistive exercise (combined treatment) compared to aerobic training on the BP, body composition and insulin sensitivity in hypertensive older adults receiving drug therapy.

## MATERIALS AND METHODS

The study was approved by the Research Ethics Committee of the Faculty of Medicine of Ribeirão Preto (protocol N° Public Health. 367/CEP/CSE-FMRP-USP).

We selected older adults, aged 60 to 75 years, who were regularly using antihypertensive medication (hydrochlorothiazide, angiotensin converting enzyme inhibitors or angiotensin II receptor blockers). The exclusion criteria were the following: mean BP above 160 mmHg for systolic blood pressure (SBP) and 105 mmHg for diastolic blood pressure (DBP); use of beta-blockers or presence of arrhythmias; smokers or ex-smokers who had stopped smoking within less than 5 years; presence of cardiac or respiratory problems that would contraindicate or limit the practice of physical exercise; ethanol intake of more than 168 g per week; a history or previous diagnosis of acute myocardial infarction, cerebrovascular accidents, or secondary hypertension due to renal injury, diabetes mellitus, degenerative cognitive changes, uncontrolled hypothyroidism and hyperthyroidism; hemoglobin values less than 11 g/d; alterations in the ergometric test or echocardiogram; limiting osteoarthritis; obesity grades II and III; peripheral vascular obstruction; enrollment in other programs or practicing regular physical activity more than twice a week; or unwilling to participate in the study.

### Pre-intervention assessment and selection procedures

Based on the selection criteria described above, 90 hypertensive older adults gave written informed consent and were assessed by applying a structured questionnaire and laboratory tests (hematology, fasting glycemia, glycated hemoglobin, creatinine, urea, sodium, potassium, TSH, and routine urinalysis).

Individuals with a glycated hemoglobin of 6.5% or higher were excluded. Eye fundoscopy and echocardiogram were performed to detect retinal diseases and cardiac hypertrophy that might prevent safe participation.

Body composition data were obtained, including the weight, height, waist circumference (WC), abdominal circumference (AC), body mass index (BMI) and fat percent determined by body bioimpedance (BIA 101Q, RJL Systems, Detroit, MI, USA).

The ergometric test was performed during the week preceding training to exclude patients with detected ischemia and to facilitate patient classification as active or sedentary based on maximum oxygen consumption (VO_2_ max). Based on these values, we determined the category to which each patient belonged according to tables 11 of aerobic conditioning for men and women.

BP was initially measured using an oscillometric device (OMRON-HEM 4031, Dalian Economic and Technical Development Zone, CHINA).

Of the 90 older adults who were assessed, 46 were excluded based on one or more of the established exclusion criteria and 44 were included in the study.

### Study protocol

After the initial general assessment, the participants underwent 24-hour ambulatory blood pressure monitoring (ABPM) (SPACELABS Medical, model 90207, Issaquah, WA, USA) on the non-dominant arm.

The oral glucose tolerance test (OGTT) was performed with insulin measurements. The degree of insulin sensitivity (%IS) was estimated by homeostasis model assessment (HOMA) 12, the insulin sensitivity index (IS) 13,14, and calculation of the area under the curve (AUC) for glucose and for insulin after the oral glucose load 15. Insulin sensitivity was determined as the ratio of the insulin AUC to the glucose AUC 15.

The volunteers were randomized to the following three groups using the SAS system, version 9 (SAS Institute, Cary, NC, USA): resistance and aerobic training (RAG), aerobic training (AG), and control (CG).

The training protocol lasted 10 consecutive weeks with three sessions per week on alternate days for 30 uninterrupted sessions.

Before each training session, the subjects remained at rest for 15 minutes for the measurement of BP (OMRON – HEM 4031) and heart rate (HR) with a 5-minute interval between measurements.

AG subjects trained on a treadmill ergometer. The first 5 minutes of each session were devoted to warm-up exercises, followed by 20 minutes of continuous aerobic exercise (AE) from the first to the 4th week. The time was increased to 30 minutes of continuous AE from the fifth to the 10th week. Exercise intensity was based on the physical conditioning of each participant, and subjects were stimulated to remain within the training range.

RAG subjects first performed the resistive exercise (RE) followed by AE. The one maximum repetition test (1RM) was applied individually to determine the RE load. The individuals first warmed up and then completed one round of the circuit, consisting of the following 9 exercises: leg press 45°, bench press, extensor bench, handle front, flexor bench-seated (adapted), upright row, plantar flexion, seated row, and abdominals. From the first to the 4th week, the volunteers completed one circuit round. During the 5th week, the 1RM was recalculated and one circuit round was added, giving a total of two rounds of the same circuit performed in the same sequence from the 5th to the 10th week. 

The time between stations (each device) was 1 minute. Each station consisted of exercises performed in a series of 15 repetitions for the upper limbs and 20 repetitions for the trunk and lower limbs. The intensity was determined individually and training was performed with 50 to 60% 1RM obtained in the test.

After execution of the RE protocol, AE was performed in the same manner as for the AG.

CG subjects did not perform any type of training; instead, they maintained their normal daily activities throughout the study period.

### Post-intervention procedures

ABPM was installed 24 hours after the 10th session for each group. All participants belonging to each group underwent a new OGTT with insulin determination, anthropometric measurements, and a new test for the determination of maximum aerobic power (ergometric test). All tests were perormed in a rigorously identical manner to those performed during the pre-intervention period.

### Statistical analysis

Data were analyzed using the mixed effects linear model, which is appropriate for situations in which the data do not show normal distribution, and the response variables underwent logarithmic transformation.

The Wilcoxon test was used to determine the presence or absence of an effect of the intervention (pre- and post-training) for each group while considering the AUC for glycemia and insulin.

Analysis with delta calculation was used to determine the magnitude of the effect of training. All analyses were performed with SAS^®^ 9.0 software.

## RESULTS

Table 1 presents the characteristics of the studied groups. There was no difference in the variables between groups (*p*>0.05). The initial workload values for each station were leg press: 77.6±36.5 kg; bench press: 14.7±5.9 kg; extensor bench: 11.8±4.8 kg; handle front: 24.2±8.3 kg; flexor bench seated: 11.8±6.1 kg; upright row: 27.2±7.7 kg; plantar flexion: 10.5±5.0 kg, and seated row: 27±8.7 kg.

This load was significantly increased for leg press (*p*=0.009) and plantar flexion (*p*=0.003) with mean values of 102.7±44.1 kg and 17.4±7.5 kg after the 5th week and mean values of 128.8±48 kg and 20.5±9.4 kg after 10 weeks, respectively.

The VO_2_ changed significantly in the AG and RAG in the pre- and post-treatment comparison, which had initial values of 25.2±7.2 and 28.2±9.1 ml/kg/min and final values of 33.1±4.2 and 34.9±7.9 ml/kg/min, respectively (*p*<0.001).

**Figure 1:**
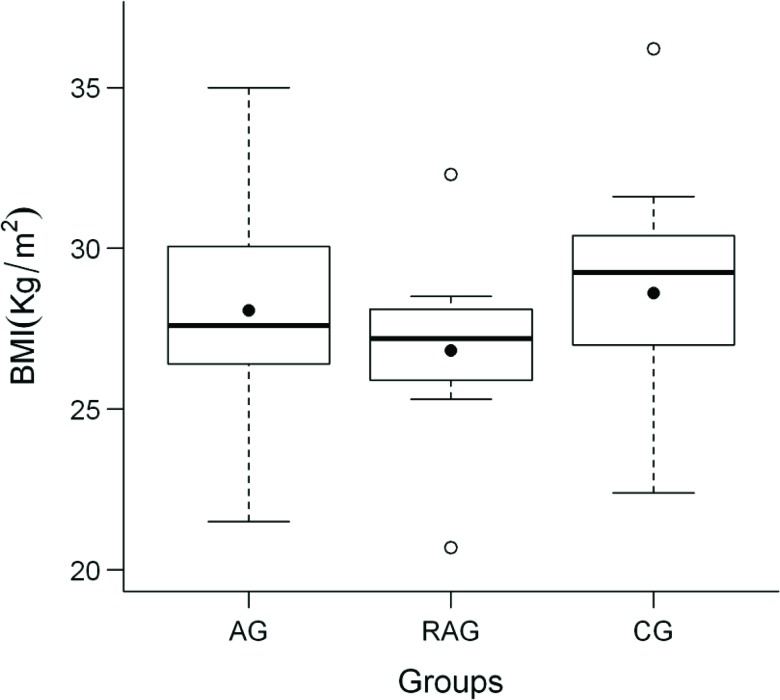
Body mass index (BMI) before and after treatment for the aerobic training (AG), resistance and aerobic training (RAG) and control (CG) groups.

The pre-training BMI values were similar for the three groups and then decreased in the AG and RAG and increased in the CG (*p*<0.001) observed after training (Figure 1).

However, fat mass was only modified after training in the RAG compared to the CG (*p*=0.01), and there was no change for the AG or CG (Table 2). No change in lean mass was observed in the three groups (Table 2).

AC was reduced after training for both the AG and RAG (*p*<0.01), whereas CG values tended to increase at the end of the observation period (*p*=0.07) (Table 2). The same was observed for WC, whose values were reduced for the AG and RAG and increased for the CG (Table 2).

Regarding the pressure values obtained by ABPM, the AG and RAG showed a decrease in the systolic 24-hour BP (SBP) from the pre- to the post-training periods (*p*=0.02 and *p*<0.001, respectively) and CG showed a slight increase in the BP (*p*>0.001) (Figure 2a). The magnitude of the decrease was similar for the AG and RAG. The same trend was observed for the SBP obtained during wakefulness along time for the AG (*p*=0.01) and RAG (*p*<0.01), which increased for the CG (*p*=0.01). During sleep, there was a decrease in the SBP in the AG and RAG (*p*<0.01), while there was an increase in the SBP in the CG (*p*=0.04) (Table 3).

The twenty-four-hour DBP had a significant decrease for the AG and RAG (*p*<0.01) in the pre- and post-training comparison (Figure 2b). During wakefulness, there was a reduction of 4 mmHg (*p*<0.01) in the AG and RAG, with no difference for the CG (Table 3). The sleep DBP was similar before and after the intervention for the three groups, whereas the comparison between time points decreased in the AG (*p*<0.01) and RAG (*p*=0.01) groups (Table 3).

**Figure 3:**
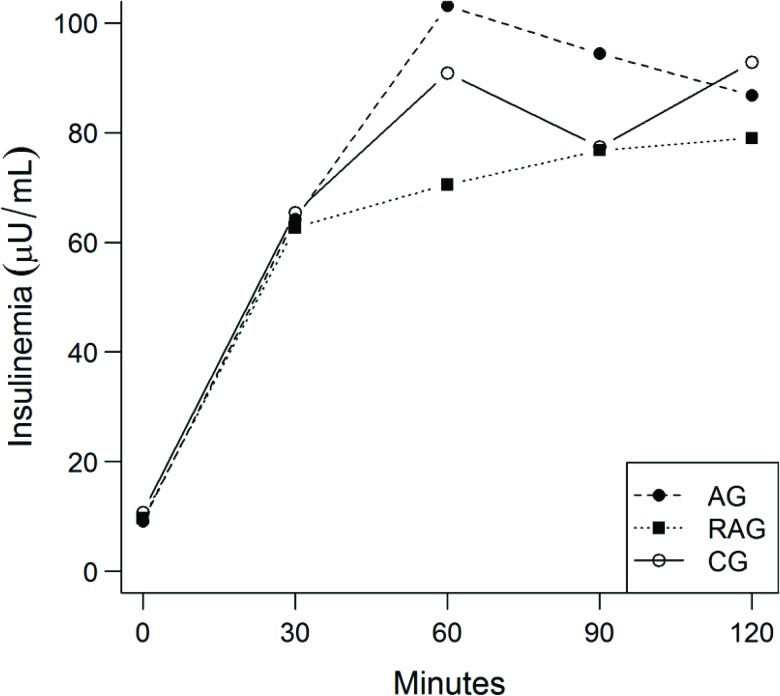
Insulinemia with time for the aerobic training group (AG), resistance and aerobic training group (RAG) and control group (CG).

The AUC for glucose (Table 4) and AUC for insulin (Table 4 and Figure 3) did not differ between the groups with time. The ratio of the two curves did not differ between treatments (*p*=0.34). Again, no difference was observed in the HOMA IR (*p*=0.94), HOMA beta (*p*=0.81) or the sensitivity index calculated from the OGTT (*p*=0.24) (Table 4).

No cardiovascular intervention was observed during the exercises nor did we detect orthopedic lesions or pain after the training sessions.

## DISCUSSION

In the present study, only 44 of the 90 recruited older adults were fit to perform physical activity because our selection criteria were rigorous, excluding individuals with diseases or factors that are commonly detected in the older adult population, such as excessive alcohol consumption, obesity, diabetes, smoking, and the use of beta blockers, among others. However, it would have been difficult to monitor the hemodynamic and metabolic effects if the study group had been heterogeneous. In light of the above considerations, we attempted to obtain the most homogeneous and reproducible sample possible.

Muscle strength and power are important markers of morbidity and mortality 16. In the present study, older adults who underwent resistive training significantly increased their work load by 65% on the leg press and by 95% in plantar flexion after the 10 weeks of training. In a recent study 17,18, the authors detected an increase in muscle strength in elderly subjects after 16 weeks of combined aerobic and anaerobic training.

In addition to the changes in muscle mass and strength due to aging, there seems to be an inevitable decline in the cardiorespiratory fitness, which can be observed in parallel to the decline in VO_2_. In the present study, the VO_2_ values increased by 23.8% for the AG and by 19.2% for the RAG in the pre- and post-training comparison. This result is clinically important because, according to the literature, a 10% increase in the VO_2_ can result in a 15% reduction in the cardiovascular mortality 19. This elevation in the VO_2_ has also been reported in other studies 20,21.

Regarding body composition, a significant decrease in the BMI values was observed in both AG and RAG compared to CG. Similar results were obtained in a study 22 in which elderly individuals who underwent 12 weeks of RE achieved an expressive reduction of BMI. Similar effects were observed after a 12-week exercise program in postmenopausal women 23. Different results have been reported 24 for older adults who underwent aerobic and resistive exercise; they exhibited no changes in body weight or BMI after 12 weeks of training.

In the present study, the fat mass was only reduced in RAG with time, and there was no change in the AG or CG fat mass. Similar findings were obtained in another study 25 that reported only older adults who underwent combined training achieved effective fat mass reduction compared to AE and control subjects.

Similarly, and as expected, the AC measures were smaller after training compared to the CG. Reduction of the BMI, total abdominal fat and subcutaneous and visceral fat was observed after six months of aerobic and resistance training in a sample of 115 older adults 26.

In the present study, the 24-hour SBP and DBP were reduced by 4.2/3.4 mmHg for the AG and by 7.7/3.8 mmHg for the RAG and they were increased by 5.7/0.7 mmHg in the CG in the pre- and post-training comparison, respectively. Maintenance of SBP reductions of 2 mmHg would result in a 6% reduction of mortality due to cerebrovascular accidents and a 4% reduction of mortality due to coronary artery disease 27.

The present data agree with previous studies that demonstrated a decrease in BP after AE 28. On the other hand, a study involving moderate intensity at a frequency of three times a week, over a period of 9 months, observed a more impressive decrease in BP after combined training compared to aerobic training alone 29. However, only three BP measurements were obtained in a single morning in that study.

The DBP was less responsive than the SBP, although it had significant reductions after the program for the AG and RAG. Several investigators have also reported a larger decrease in the SBP 30,31 than in the DBP.

BP reductions were also observed when only the wakefulness or sleep period was assessed for the SBP and DBP in the AG and RAG, which agrees with some other studies 32,33 and disagrees with others 34.

No change in the IS-OGTT, insulin AUC/glucose AUC, HOMA IR or HOMA Beta was observed with time. However, the insulin values during the GTT were within the normal limits in the RAG alone. Starting at the 30-minute time point, the insulin values were higher than the reference values in the AG and CG.

Several studies have demonstrated improved insulin sensitivity with physical activity. According to some investigators 34, combined training improves insulin sensitivity and prevents increased glucose levels in sedentary individuals, while others 35 observed that the effects of combined training were indistinguishable from those of aerobic training alone in volunteers aged 18 to 70 years for a period of 8 months. In another study 36, a reduction in the AUC for glucose and insulin was observed in the AG, while only a reduction in the AUC for insulin was observed in the RAG. However, insulin sensitivity increased in the RAG and AG. The study was conducted on middle-aged individuals over a period of 12 weeks, which may explain the difference in results compared to the present study. The period of 10 weeks was possibly insufficient to modify insulin sensitivity to a significant degree.

In conclusion, the group that underwent combined aerobic and resistance training did not show a greater risk when performing the exercises based on the observed decreases in BP demonstrated with the 24-hour ABPM. However, there was no greater decrease in the BP after combined resisted and aerobic training. Improvement of aerobic capacity and reduction of the BMI, WC and AC were also similar between the combined exercise and AE alone groups. The fat mass was only significantly reduced with combined aerobic and resistance training. Because resistance training has proven benefits for maintaining functionality in older adults, the present findings support the use of combined exercise in elderly hypertensive patients without impairment of the BP response.

These results confirm that there were no additional benefits regarding the decrease in the 24-hour blood pressure with combined aerobic and resistance training versus aerobic training alone in hypertensive older adults. No other study has evaluated blood pressure by ambulatory blood pressure monitoring (the gold standard method) before and after aerobic training alone and combined exercise in hypertensive older individuals. However, the fat mass was only reduced with combined training, and there were no adverse cardiovascular effects during exercise. This study endorses the use of combined training in hypertensive older adults.

## AUTHOR CONTRIBUTIONS

All authors contributed significantly to this study and have read and approved the submitted manuscript. Lima LG designed and conducted the research and wrote the manuscript. Bonardi JT, Campos GO, Bertani RF and Scher LM conducted the research. Moriguti JC and Ferriolli E designed the research and analyzed the data. Lima NK designed the research, analyzed the data, wrote the manuscript and had primary responsibility for final content.

## Figures and Tables

**Figure 2a:**
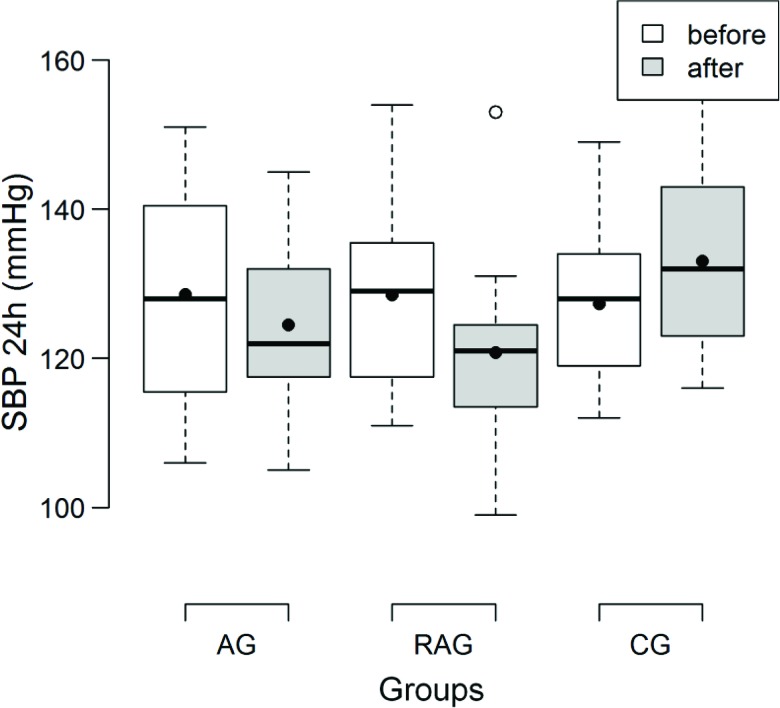
Box plot of the mean systolic blood pressure (SBP) (mmHg) obtained by 24-hour ambulatory arterial blood pressure monitoring before and after all sessions for the aerobic training (AG), resistance and aerobic training (RAG) and control (CG) groups.

**Figure 2b:**
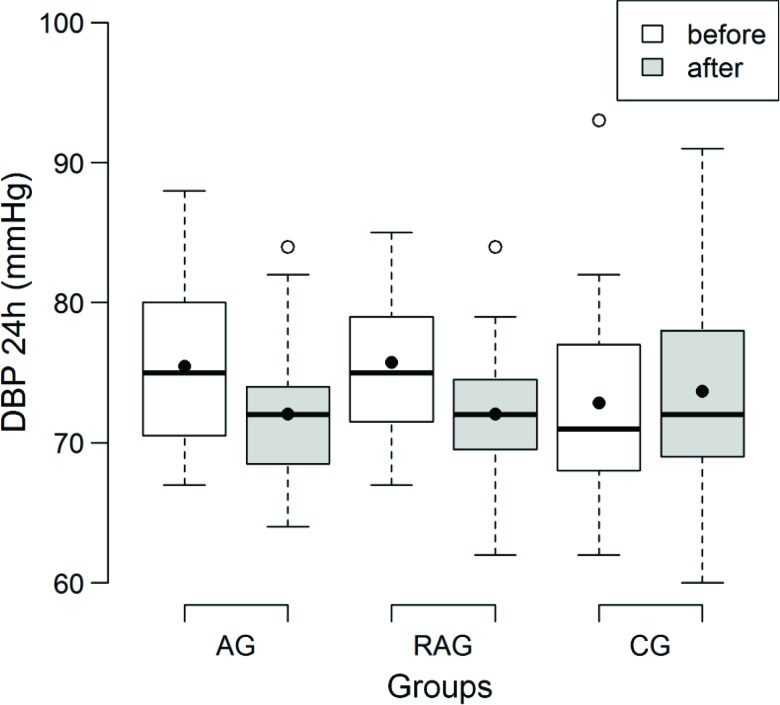
Box plot of the mean 24-hour diastolic blood pressure (DBP) (mmHg) obtained by 24-hour ambulatory arterial blood pressure monitoring before and after all sessions for the aerobic training (AG), resistance and aerobic training (RAG) and control (CG) groups.

**Table 1:** Clinical characteristics of the groups before the training period.

Characteristics	AG	RAG	CG
**N**	15	15	14
**Age (years)**	67.8±4.3	67.8±5.2	69.9±5.5
**Sex (male/female)**	1/14	4/11	2/12
**BMI (kg/m^2^)**	28.9±3.5	28±3.2	27.6±3.4
**Metabolic rate (cal/day)**	1350.0±236.4	1361.6±263.2	1359.2±284.9
**VO_2_**max (ml/kg/min)	25.2±7.2	28.2±9.1	28.3±8.5
**AC (cm)**	96.1±8.5	92.4±5.5	91.7±11.4
**WC (cm)**	90.1±12	88.9±7.7	86.1±11
**Lean mass (kg)**	42.2±7.8	44.3±8.3	43.4±9.3
**Fat mass (kg)**	26.2±5.8	24.5±5.9	24.6±6.3
**24-h SBP (mmHg)**	128.6±14.4	128.5±11.7	127.3±11
**24-h DBP (mmHg)**	75.4±6.0	75.7±5.5	72.8±8.1
**SBP sleep (mmHg)**	123.0±15.0	124.2±16	123.6±12.3
**DBP sleep (mmHg)**	69.4±7.9	71.7±9.2	67.6±7.6
**SBP awake (mmHg)**	132.2±14.4	130.3±9.6	130.3±11.7
**DBP awake (mmHg)**	79±5.7	77.9±4.6	75.5±8.5
**Heart rate (bpm)**	72.8±8.5	77.3±11.6	72.6±6.5

Values are reported as the mean ± SD. AG - aerobic training group; RAG - </underline>resistance and aerobic training group; CG - control group; N - number; BMI - body mass index; VO_2_max - maximum oxygen consumption; AC - abdominal circumference; WC - waist circumference; 24-h SBP - 24-h systolic blood pressure with ambulatory blood pressure monitoring; and 24-h DBP - 24-h diastolic blood pressure with ambulatory blood pressure monitoring.

**Table 2:** Body composition and anthropometric measurements before (Pre) and after (Post) training

Variables		Groups	Mean	SD
**Fat mass (kg)**	Pre	AG	26.7	5.8
		RAG	24.5	5.9
		CG	24.6	6.3
	Post	AG	25.4	5.6
		RAG	21.8*	3.9
		CG	26.1	6.4
**Lean mass (kg)**	Pre	AG	42.2	7.8
		RAG	44.3	8.3
		CG	43.4	9.3
	Post	AG	41.9	6.6
		RAG	44.1	6.9
		CG	43.4	9.0
**Abdominal circumference (cm)**	Pre	AG	96.1**	8.5
		RAG	92.4**	5.5
		CG	91.7	11.4
	Post	AG	92.9	8.6
		RAG	89.4	6.1
		CG	92.9	11.6
**Waist circumference (cm)**	Pre	AG	90.1**	12
		RAG	88.9**	7.7
		CG	86.1	11
	Post	AG	88.5	11.9
		RAG	86.7	7.6
		CG	87.2	11.6

The mean and standard deviation (SD) for the variables fat mass, lean mass, abdominal circumference and waist circumference before and after sessions for the aerobic training group (AG, n=15), resistance and aerobic training group (RAG, n=15) and control group (CG, n=14).

* *p*=0.01 (RAG *vs*. CG) and

** *p*< 0.01 (pre *vs*. post).

**Table 3:** Systolic and diastolic blood pressures of the various groups during wakefulness and sleep.

Variables		Groups	Mean	SD
**SBP awake**	Pre	AG	132.2**	14.4
		RAG	130.3**	9.6
		CG	130.3**	11.7
	Post	AG	127.4	12.1
		RAG	122.8*	9.9
		CG	135.3	12.1
**SBP sleep**	Pre	AG	123**	15.3
		RAG	124.2**	16
		CG	123.6**	12.3
	Post	AG	117.2	11
		RAG	117.6	16.5
		CG	128.1	13.5
**DBP awake**	Pre	AG	79.0**	5.7
		RAG	77.9**	4.6
		CG	75.5	8.5
	Post	AG	74.5	5.6
		RAG	74.4	4.9
		CG	77.3	8.0
**DBP sleep**	Pre	AG	69.4**	7.9
		RAG	71.7**	9.2
		CG	67.6	7.6
	Post	AG	65.5	6.7
		RAG	68.0	7.6
		CG	67.9	8.6

The mean and standard deviation (SD) for the variables systolic blood pressure (SBP) and diastolic blood pressure (DBP) during awake periods and sleep before (pre) and after (post) sessions for the aerobic training group (AG), resistance and aerobic training group (RAG) and control group (CG).

* *p*<0.05 (RAG *vs*. CG) and

** *p*< 0.05 (pre *vs*. post).

**Table 4:** Assessment of insulin sensitivity before (Pre) and after (Post) training

Variables		Groups	Mean	SD
**AUC-Gluc (mg/dL)**	Pre	AG	318.1	48.4
		RAG	270.0	47.7
		CG	294.9	55.5
	Post	AG	311.6	45.4
		RAG	262.1	51.2
		CG	308.4	70.2
**AUC-Ins (μU/ml)**	Pre	AG	123.4	67.9
		RAG	131.5	72.7
		ACG	105.5	45.7
	Post	AG	154.8	141.9
		RAG	127.1	85.2
		CG	140.3	78.2
**IS-OGTT (μIU/ml)**	Pre	AG	0.20	0.01
		RAG	0.20	0.01
		CG	0.20	0.01
	Post	AG	0.20	0.01
		RAG	0.20	0.02
		CG	0.20	0.01
**HOMA IR (mMol/L.µU/ml)**	Pre	AG	1.79	0.87
		RAG	1.67	1.03
		CG	1.95	0.96
	Post	AG	2.20	1.38
		RAG	2.17	1.31
		CG	2.50	1.52
**HOMA BETA (%)**	Pre	AG	91.9	31.6
		RAG	25.2	55.2
		CG	114.2	58.2
	Post	AG	120.0	55.7
		RAG	140.8	60.3
		CG	133.8	116.0

Values are reported as the mean±SD. AUC - area under the curve, Ins - insulin, gluc - glucose, OGTT - oral glucose tolerance test, IS-OGGT - Bastard index, HOMA - Homeostasis assessment, AG - aerobic training group, RAG - resistance and aerobic training group, and CG - control group.
